# The Effect of a Life-Style Intervention Program of Diet and Exercise on Irisin and FGF-21 Concentrations in Children and Adolescents with Overweight and Obesity

**DOI:** 10.3390/nu13041274

**Published:** 2021-04-13

**Authors:** Sofia I. Karampatsou, Sofia M. Genitsaridi, Athanasios Michos, Eleni Kourkouni, Georgia Kourlaba, Penio Kassari, Yannis Manios, Evangelia Charmandari

**Affiliations:** 1 Division of Endocrinology, Metabolism and Diabetes, First Department of Pediatrics, National and Kapodistrian University of Athens, ‘Aghia Sophia’ Children’s Hospital, 11527 Athens, Greece; karampatsousi@gmail.com (S.I.K.); sgenitsaridi@gmail.com (S.M.G.); peniokassari@gmail.com (P.K.); 2 Division of Infectious Diseases, First Department of Pediatrics, National and Kapodistrian University of Athens, ‘Aghia Sophia’ Children’s Hospital, 11527 Athens, Greece; athanmic@yahoo.com; 3 Center for Clinical Epidemiology and Outcomes Research (CLEO), 11528 Athens, Greece; e.kourkouni@cleoresearch.org (E.K.); kurlaba@gmail.com (G.K.); 4 Department of Nutrition and Dietetics, Harokopio University of Athens, Kallithea, 17671 Athens, Greece; manios@hua.gr; 5 Division of Endocrinology and Metabolism, Center of Clinical, Experimental Surgery and Translational Research, Biomedical Research Foundation of the Academy of Athens, 11527 Athens, Greece

**Keywords:** irisin, FGF-21, overweight, obesity, childhood, adolescence

## Abstract

Overweight and obesity in childhood and adolescence represent major public health problems of our century, and account for increased morbidity and mortality in adult life. Irisin and Fibroblast Growth Factor 21 (FGF-21) have been proposed as prognostic and/or diagnostic biomarkers in subjects with obesity and metabolic syndrome, because they increase earlier than other traditional biomarkers. We determined the concentrations of Irisin and FGF-21 in children and adolescents with overweight and obesity before and after one year of a life-style intervention program of diet and physical exercise and explored the impact of body mass index (BMI) reduction on the concentrations of Irisin, FGF-21 and other cardiometabolic risk factors. Three hundred and ten (*n* = 310) children and adolescents (mean age ± SD: 10.5 ± 2.9 years) were studied prospectively. Following one year of the life-style intervention program, there was a significant decrease in BMI (*p* = 0.001), waist-to-hip ratio (*p* = 0.024), waist-to-height ratio (*p* = 0.024), and Irisin concentrations (*p* = 0.001), and an improvement in cardiometabolic risk factors. There was no alteration in FGF-21 concentrations. These findings indicate that Irisin concentrations decreased significantly as a result of BMI reduction in children and adolescents with overweight and obesity. Further studies are required to investigate the potential role of Irisin as a biomarker for monitoring the response to lifestyle interventions and for predicting the development of cardiometabolic risk factors.

## 1. Introduction

Overweight and obesity in childhood and adolescence represent major public health problems of our century owing to their increased prevalence during the last four decades and their association with increased morbidity and mortality in adult life [1]. The World Health Organization (WHO) has reported that 41 million children younger than 5 years and 340 million children and adolescents aged 5–19 years have overweight or obesity, while 124 million children and adolescents have obesity worldwide [2,3]. Obesity accounts for approximately 5% of all deaths worldwide, as well as for a significant increase in public health costs [4]. The global economic impact from obesity is approximately $2.0 trillion USD or 2.8% of the global gross domestic product (GDP), which is almost equivalent to the global impact from smoking or armed violence, war, and terrorism [4]. In Greece, the prevalence of overweight and obesity is one of the highest among European countries [5].

Obesity is defined by the body mass index (BMI) and is characterized by an increase in the adipose tissue [6], and the release of pro-inflammatory, atherogenic cytokines, which predispose to the development of cardiometabolic risk factors and atherosclerotic cardiovascular decease later in life [7].

In order to address the epidemic of childhood obesity, to predict the risk of development of cardiometabolic abnormalities, and to evaluate the response to lifestyle and therapeutic interventions, novel biomarkers, such as Irisin and Fibroblast Growth Factor 21 (FGF-21), have been proposed as prognostic and diagnostic markers in adults and children with overweight and obesity [8,9,10,11,12,13,14,15,16,17,18,19,20].

Irisin is a myokine discovered by Bostrom et al. as the cleavage of the N-terminal of fibronectin type III domain-containing protein 5 (FNDC5) [8]. The protein took its name from the Greek goddess Iris, who transferred messages from gods to people. Similarly, Irisin facilitates the communication between muscle and adipose tissue [8]. Irisin is released from the muscle mass during physical activity and fasting, as well as from the adipose tissue and liver [8,9]. The expression of Irisin is regulated by the coactivator a_1 (PGC-a1), a protein produced by muscle tissue during exercise, which promotes the expression of uncoupling protein 1 (UCP1) from the brown adipose tissue (BAT) [8]. Interestingly, it is transcriptionally identical in humans and mice, suggesting that it plays a pivotal role in the evolution of species [8]. Irisin has endocrine and autocrine functions, and its main role is the conversion of white adipose tissue (WAT) to brown adipose tissue (BAT) [8,9]. In addition, it improves glucose homeostasis and lipid profile [10], although elevated Irisin concentrations have been associated with decreased insulin sensitivity [11,12], atherosclerosis [13,14], and increased risk of metabolic syndrome [15] in children and adults. On the other hand, children who are underweight demonstrate lower Irisin concentrations [16]. Irisin has been proposed as a potential biomarker in adults with metabolic syndrome, because it increases much earlier than any other alterations are noted in the traditional biomarkers [17]. In children with obesity, Irisin may also serve as an early biomarker of metabolic syndrome, with a cut-off point of 44.75 ng/mL having 70.0% sensitivity and 60.0% selectivity [11].

FGF-21 belongs to the human Fibroblast Growth Factor (FGF) family. It was first described by Nishimura et al. in 2000 as a protein that belongs to FGF-19 subfamily and is expressed in the liver [18,19]. In order to function as a hormone, FGF-21 needs the transmembrane co-receptor Klotho to activate FGF receptors [20,21]. Except for the liver, FGF-21 is also secreted from the skeletal muscle, pancreas, WAT, and BAT [15,18], and has endocrine, paracrine, and autocrine actions [22,23]. It regulates glucose and lipid metabolism [24,25], and activates thermogenesis in response to exposure to cold [23]. Similar to Irisin, FGF-21 increases the expression of UCP1 in order to activate the BAT and the browning of WAT, and to increase the thermogenic capacity of the organism [23,26]. Moreover, FGF-21 protects against non-alcoholic fatty liver disease (NAFLD) and the stress caused by the metabolic disorders in the liver [19,25]. In children with obesity, FGF-21 decreases after the implementation of an intervention program of weight loss [27], while in adolescents with diabetes mellitus type 2 (DM2), FGF-21 concentrations are higher than in those without DM2 [28]. Finally, administration of recombinant FGF-21 in rodents and humans improves the lipid profile and insulin sensitivity, indicating that this analogue may be useful in the pharmaceutical management of the complications of obesity [29,30] and insulin resistance [31]. FGF-21 concentrations are increased in metabolic syndrome and are independently related to homeostasis model assessment of insulin resistance (HOMA-IR) [31,32]. Furthermore, in rats fed with high-fat diet, FGF-21 increases prior to the development of insulin resistance. As a result, FGF-21 may also serve as a biomarker indicating increased risk for the development of metabolic syndrome [33]. However, available studies investigating the effect of diet and/or exercise on Irisin and FGF-21 concentrations in childhood and adolescence are few [27,34,35,36,37,38,39].

The aim of our study was to determine the concentrations of Irisin and FGF-21 in children and adolescents with overweight and obesity before and after one year of a life-style intervention program of diet and physical exercise, and to explore the impact of BMI reduction on the concentrations of Irisin, FGF-21, and other cardiometabolic risk factors in these subjects. The novelty of our study consists mainly in (i) the larger sample of children and adolescents studied prospectively; (ii) the implementation of a comprehensive, multidisciplinary, and personalized intervention program, which was well-designed and executed by experienced professionals; and (iii) the longer duration of the follow-up period.

## 2. Materials and Methods

### 2.1. Patients

Three hundred and ten (*n* = 310) children and adolescents (mean age ± SD: 10.5 ± 2.9 years; 162 males, 148 females; 152 prepubertal, 158 pubertal), aged 2–18 years old, attending our Out-patient Clinic for the Prevention and Management of Overweight and Obesity in Childhood and Adolescence, "Aghia Sophia" Children’s Hospital, Athens, Greece, were studied prospectively for 1 year. Subjects were classified as having obesity *(n* = 211, 68%), if their BMI was above the 95th percentile for age and gender, or as having overweight (*n* = 99, 32%) if their BMI was between the 85th and 95th percentile for age and gender, according to the International Obesity Task Force (IOTF) criteria [40,41]. Patients were included in the study if they were 2–18 years old and had increased BMI for their age and gender, according to the IOTF criteria [40]. They were excluded from the study if they had syndromic obesity, i.e., obesity that is inherited through mendelian patterns and is associated with other clinical manifestations [42]. The clinical characteristics of all subjects are summarized in Table 1. 

#### 2.1.1. Methods

All subjects were admitted to our Out-patient Clinic for the Prevention and Management of Overweight and Obesity in Childhood and Adolescence early in the morning on the day of the study. A single trained observer obtained a detailed medical history and all standard anthropometric measurements (weight, height, waist circumference, hip circumference), and performed a thorough clinical examination, including pubertal assessment. Body weight and standing height were measured in light clothing and without shoes using, respectively, the same scale (Seca GmbH and Co. KG., Hamburg, Germany) and the same Harpenden’s stadiometer (Holtain Limited, Crymych-Dyfed, UK) in all subjects. Waist and hip circumferences were measured according to WHO STEPS protocol using the same stretch-resistant tape (Seca GmbH and Co. KG., Hamburg, Germany) [43]. Systolic (SBP) and diastolic blood pressure (DBP) was determined twice (sphygmomanometer Comfort 20/40, Visomat, Parapharm, Metamorphosi, Attiki, Greece) and the mean value was calculated. Moreover, all subjects underwent bioelectrical impedance analysis (BIA) (TANITA MC-780U Multi Frequency Segmental Body Composition Analyzer, Amsterdam, The Netherlands). Baseline hematologic, biochemical and endocrinologic investigations were performed at 08:00 h after a 12 h overnight fast.

All subjects were evaluated by a Pediatrician, Pediatric Endocrinologist, Pediatric Dietician, and a professional fitness Personal Trainer, and entered a personalized, comprehensive, multidisciplinary management intervention program that provided personalized advice and guidance on diet and physical exercise to patients and their families for one year [44]. Subjects with obesity were followed up every month and subjects with overweight every two months. Upon each follow-up visit, all anthropometric measurements were determined, and the goals set in previous sessions were discussed in detail with the Pediatric Dietitian and Personal Trainer. Detailed hematologic, biochemical, and endocrinologic investigations were performed one year later, at the end of the study, at 08:00 h following a 12 h overnight fast. All subjects included in the study complied with the advice given on diet and exercise. The compliance was ascertained by the Pediatrician according to the achieved goals set in the previous session, as well as the decreased in BMI (at least 0.6 kg/m^2^) or BMI z-score [45] at the end of the study.

The Pediatric Dietician evaluated the daily nutritional habits of all subjects, performed a 24 h recall according to the USDA method [46,47], and recommended a personalized plan of healthy diet, taking into account both the child’s preferences and the food availability at school or at home. The Personal Trainer recommended a personalized physical exercise plan, which was considered enjoyable and entertaining.

#### 2.1.2. Assays

Standard hematologic, biochemical [glucose, HbA1C, total cholesterol, HDL, LDL, triglycerides, apolipoprotein A1 (ApoA1), apolipoprotein B (ApoB), Lipoprotein (a) (Lp(a)), high sensitivity C-reactive protein (hsCRP)] and endocrinologic [TSH, T3, Free T4, anti-TPO, anti-TG, insulin, ferritin, total 25-hydroxyvitamin D (25-OH Vitamin D)] investigations were determined as previously described [44].

Irisin concentrations were determined using a commercially available enzyme-linked immunoassay (ELISA) kit (Phoenix Pharmaceuticals, Burlingame, CA, USA; Cat. no. EK-067-52). The sensitivity of the assay was 4.15 ng/mL, and the assay range was 0.328–204.8 ng/mL. The intra-assay CV was <10% and the inter-assay CV was <15%.

FGF-21 concentrations were determined using a commercially available ELISA kit (Cat. No. DF2100; R and D Systems, Minneapolis, MN, USA). The sensitivity of the assay was 8.69 pg/mL, and the assay range was 31.3–2000 pg/mL. The intra-assay CV was 3.4% and the inter-assay CV was 7.5%.

Leptin concentrations were determined using an ELISA kit (Cat No. RD191001100; BioVendor R and D, Oxford Biosystems Ltd., Abington, UK). The sensitivity of the assay was 0.2 ng/mL. The intra-assay CV was 5.9% and the inter-assay CV was 5.5%.

Adiponectin concentrations were determined using an ELISA kit (Cat. No. BMS2032; eBioscience, Thermo Fisher Scientific, San Diego, CA, USA). The sensitivity of the assay was 0.01 ng/mL. The intra-assay CV was 4.2% and the inter-assay CV: 3.1%.

Insulin resistance (IR) was determined according to the homeostasis model assessment (HOMA) as follows: HOMA-IR = (fasting glucose (mg/dL) × fasting insulin (mU/L))/405. Tri-ponderal mass index (TMI) was calculated using the formula: TMI = mass divided by height cubed.

#### 2.1.3. Statistical Analysis

Nominal variables are presented with absolute and relative frequencies (%), while continuous variables with mean, standard deviation (SD), median, and interquartile range. Normality of continuous variables was performed graphically with histograms. Differences between BMI categories and patients’ baseline characteristics were evaluated with X^2^ test of independence and *t*-test or Mann–Whitney test. In order to detect any changes in anthropometric and body composition parameters, as well as hematologic, biochemical and endocrinologic measurements after the 12-month intervention, we used either the Paired Samples *t*-test, or the Wilcoxon-sign rank test based on the normality of the data. The McNemar test was also performed to detect changes in qualitative measurements (e.g., BMI categories). The Mann–Whitney U test was used to detect differences in Irisin and FGF-21 concentrations according to gender and pubertal stage. Pearson’s *r* and Spearman’s rho correlation coefficients were used to check for associations between the changes in the measurements, depending on whether the data followed the normal distribution or not (e.g., association of the changes in Irisin and FGF-21 and change in cardiometabolic risk factors, such as BMI, SBP, DBP, HDL, etc.).

Multiple linear regression analysis was performed to assess the effect of changes in cardiometabolic risk factors on the changes in Irisin and FGF-21 concentrations. The factors introduced in the model as independent variables were those found to be statistically significantly associated with changes in hormone concentrations at a univariate level. Results are presented with β regression coefficients and 95% Confidence Intervals. Linear mixed models were performed to detect any differences in the changes over time between gender and pubertal stage.

The statistical significance level was set at α = 5%. Bonferroni adjustment for multiple testing was taken into consideration. All analyses were performed with the SPSS statistical package version 25.0 (SPSS Inc., Chicago, IL, USA).

## 3. Results

The study sample consisted of 310 subjects aged 2–18 years (mean age ± SD: 10.5 ± 2.9 years; 162 males, 148 females; 152 prepubertal; and 158 pubertal). The clinical characteristics of all subjects at baseline are presented in Table 1 and Table 2. Children and adolescents with obesity had significantly higher SBP (*p* = 0.035) and DBP (*p* = 0.045), waist circumference (*p* = 0.001), hip circumference (*p* = 0.001), waist-to-hip (WHR) (*p* = 0.014), and waist-to-height ratio (WHtR) (*p* = 0.001) than subjects with overweight, and these differences were all clinically significant (Table 1).

Table 2 illustrates the anthropometric, clinical, biochemical, endocrinologic, and body composition parameters in all subjects at initial and annual assessment. Following one year of life-style interventions, there was a significant decrease in BMI (*p* = 0.001) and in BMI percentile (*p* = 0.001). More specifically, the percentage of children and adolescents with obesity decreased from 27.1% to 18.1%, while the percentage of those with BMI above the 97th percentile decreased from 41% to 19.7%. The percentage of children and adolescents with overweight increased from 31.9% to 51.3% (Table 2). With respect to the body composition parameters, there was a significant decrease in fat mass (*p* = 0.001) and an increase in muscle mass (*p* = 0.001), bone mass (*p* = 0.001), and free-fat mass (*p* = 0.001) (Table 2).

In addition, following one year of the multidisciplinary management interventions, all subjects demonstrated a significant decrease in hepatic enzymes (SGOT (*p* = 0.001), SGPT (*p* = 0.001), γGT (*p* = 0.001)), total cholesterol (*p* = 0.001), LDL (*p* = 0.001) and Apo-Β (*p* = 0.001) concentrations, and a significant increase in HDL (*p* = 0.001) and total 25-OH-vitamin D (*p* = 0.001) concentrations. There was also a significant reduction in Irisin (*p* = 0.001) and Leptin (*p* = 0.001) concentrations, and a significant increase in Adiponectin (*p* = 0.001) concentrations. No statistically significant changes in FGF-21 concentrations were noted (Table 2). Furthermore, there was a significant increase in glucose (*p* = 0.006), creatinine (*p* = 0.001) and IGF-1 (*p* = 0.001) concentrations, and a significant decrease in urea (*p* = 0.001) and T3 (*p* = 0.001) concentrations; however, these parameters remained within the normal range and their changes were not clinically relevant.

FGF-21 concentrations correlated positively with BMI (*p* = 0.001), WC (*p* = 0.001), triglycerides (p = 0.001), HOMA-IR (*p* = 0.001), fat mass percentage (*p* = 0.043), muscle mass (*p* = 0.002) and bone mass (*p* = 0.003) (Table S1). 

The change in Irisin concentrations correlated negatively with the change in BMI (*r* = −0.217, *p* = 0.01), BMI z-score (*r* = −0.201, *p* = 0.001), waist circumference (*r* = −0.423, *p* = 0.001), WHR (*r* = −0.234, *p* = 0.007), PTH *(r* = −0.151, *p* = 0.012), Adiponectin *(r* = −0.161, *p* = 0.007) and TMI (*r* = −0.192, *p* = 0.001) and positively with the change in HDL (*r* = 0.232, *p* = 0.001). The change in FGF-21 concentrations correlated negatively with the change in HDL (*r* = −0.167, *p* = 0.007) and positively with the change in muscle mass percentage (*r* = 0.207, *p* = 0.003) and bone mass (*r* = 0.209, *p* = 0.003). However, most of these relations were weak, as indicated by the small rho correlation coefficients (Table 3).

The change in BMI was negatively correlated with the change in Irisin in boys (*r* = −0.302, *p* = 0.001) and in girls (*r* = −0.302, *p* = 0.001). Moreover, the change in BMI was negatively correlated with the change in Irisin concentrations in pubertal (*r* = −0.265, *p* = 0.001) but not in prepubertal subjects. Finally, the change in BMI correlated negatively with the change in Irisin concentrations only in subjects with obesity (*r* = −0.407, *p* = 0.001) but not in subjects with overweight. No correlation was found between the change in BMI and the change in FGF-21 concentrations (Table 4).

Multivariate linear regression analysis indicated that the change in WHR was the only independent variable affecting Irisin change (β = −465.55 (95% CI: −918.40 to 12.70), *p* = 0.044). As a result, a 0.1 increase in the change of WHR causes a 46.5 points decrease in the change of Irisin (Table 5). No independent variable was found to affect the change of FGF-21 concentrations. All variables listed in Table 5 were included in the multivariate linear regression analysis, and the model was adjusted for age, gender, and pubertal status.

## 4. Discussion

In our study, we determined the Irisin and FGF-21 concentrations in children and adolescents with overweight and obesity before and one year after the implementation of a personalized, comprehensive, and multi-disciplinary life-style intervention program of diet and physical exercise. We demonstrated that the decrease in BMI resulted in a reduction in Irisin concentrations, as well as an improvement in cardiometabolic risk factors, as indicated by the decrease in percentage of fat and total cholesterol, LDL, ApoB, SGOT, SGPT, and γ-GT concentrations, and an increase in HDL concentrations, percentage of muscle mass, and free-fat mass. There was no alteration in FGF-21 concentrations. To the best of our knowledge, this is the first study implementing a one-year life-style intervention program of diet and exercise, which has investigated the relation between Irisin, FGF-21, BMI, and cardiometabolic risk factors in such a large number of children and adolescents with overweight and obesity, and for such a long period of monitoring and follow-up. To further determine whether Irisin may be used as a potential biomarker, future studies are required that would include children and adolescents with obesity, overweight and normal BMI, as well as patients with and without metabolic syndrome. 

The recently discovered proteins Irisin and FGF-21 are known to be associated with obesity in adulthood; however, little is known about their role in childhood and adolescence. In our study, we demonstrated that there is a negative correlation between Irisin and adiponectin concentrations. These findings concur with those of previous studies that demonstrated an association between adiponectin with obesity, chronic inflammation, and cardiovascular risk factors, and indicate that Irisin and adiponectin may have a common regulatory mechanism with inverse actions [15,48].

We also noted a relation of Irisin concentrations with measures of adiposity and cardiometabolic risk factors. More specifically, the change of Irisin concentrations correlated negatively with the change in waist circumference, WHR and TMI, and positively with the change in HDL concentrations. Interestingly, the multivariate linear regression analysis revealed that the change of WHR is the strongest determinant of the change of Irisin: 0.1 increase in the change of WHR causes a 46.5 points decrease in the change of Irisin. WHR represents an alternative measure of adiposity and cardiometabolic risk [49,50]. Several studies reported similar findings in both children and adults. In adults, Irisin has been shown to be a predictor of CVD [13,15]. In children with obesity, dietary intake rich in saturated fatty acids with low monounsaturated fatty acids was associated with higher Irisin concentrations [36]. In addition, there was a negative correlation between Irisin and HDL concentrations, and a positive correlation between Irisin with fasting insulin and HOMA-IR [12].

In our study, there was no correlation between Irisin concentrations and body composition parameters, such as fat mass, muscle mass, or free-fat mass. Previous studies have yielded conflicting results. In children with obesity, increased Irisin concentrations correlated positively with fat mass, but not with free-fat mass [11], while in amenorrheic athletes, there was no correlation between Irisin and fat mass; however, there was a positive association between Irisin and free-fat mass in athletes compared with non-athletes [51]. Furthermore, in adult females, there was a positive correlation between Irisin, fat mass and fat-free mass and a negative correlation with daily physical activity. Interestingly, 1 kg increase in fat mass was associated with a two-fold increase in Irisin concentrations [52]. Löffler et al. showed a positive correlation with free-fat mass in adults but not in children and an increase in Irisin concentrations immediately after a 30 min exercise, but not after long-term exercise [35]. Finally, other studies demonstrated negative correlation [16] or no correlation with physical activity [53]. These controversial findings may arise as a result of different methodologies and duration of exercise and highlight the importance of undertaking further studies to define the impact of physical activity on Irisin concentrations.

We demonstrated that Irisin concentrations were reduced at the end of our study along with the decrease in BMI. More importantly, we found that the change in BMI correlated negatively with the change in Irisin concentrations in both male and female subjects, in subjects with obesity but not in subjects with overweight, and in adolescents. Previous studies in adults and children demonstrated a positive correlation of Irisin with BMI [12,35,52], insulin resistance, and other parameters of metabolic syndrome [11,13,15]. Our findings concur with those of several studies that showed lower concentrations of Irisin in subjects with underweight compared with subjects with normal BMI or obesity [16], and no correlation of Irisin with BMI before or after a life-style intervention program implemented for one year [39]. A cut-off point of Irisin concentrations of 44.75 ng/mL was proposed by Binay et al. to differentiate between children with obesity and normal-BMI [11].

The increased Irisin concentrations in obese subjects may be owing to its release from the adipose tissue, which is increased in obesity. Pardo et al. demonstrated a positive correlation of serum Irisin concentrations with resting energy expenditure and a negative correlation with physical activity, indicating that the myokine is released from the adipose tissue rather than the muscle [52]. Accordingly, Irisin concentrations are lower in lean and underweight subjects, who have lower fat mass and percentage fat mass. A potential mechanism that explains the low Irisin concentrations in the underweight group is that Irisin stimulates the transformation of subcutaneous WAT to BAT, thereby promoting thermogenesis through uncoupling protein 1 (UCP-1) and increasing energy expenditure. UCP-1 is an enzyme that promotes oxidative phosphorylation through ATP production, resulting to energy release as heat. Thus, it is likely that the lower Irisin concentrations in children who are underweight may represent an adaptive mechanism to reserve energy [16].

FGF-21 is released from the liver [18] and participates in the glucose and lipid metabolism, having a protective role against obesity, diabetes, and NAFLD. Several studies investigated the relation between FGF-21 and body composition parameters with conflicting results. In our study, we demonstrated a positive correlation of FGF-21 concentrations with fat mass and muscle mass. In addition, the change of FGF-21 concentrations correlated positively with the change in the muscle mass but not in fat mass or free-fat mass. We also found a positive correlation between FGF-21 and age, which concurs with previous findings demonstrating an increase in FGF-21 concentrations through age groups [54,55].

FGF-21 is a regulator of metabolic homeostasis [24] during fasting and refeeding [22]. Several studies in children report a positive correlation between FGF-21, waist circumference and hip circumference [56], and total cholesterol and triglycerides [27]. In addition, FGF-21 is higher in children with obesity compared to those with normal BMI, and in children with metabolic syndrome or with diabetes mellitus type 2 [28,31,57,58]. Similarly, in our study we demonstrated a positive correlation between FGF-21 and indices of adiposity and cardiovascular risk. More specifically, FGF-21 correlated positively with waist circumference, triglycerides, and HOMA-IR.

Our study has several strengths. Firstly, the comprehensive, multidisciplinary, personalized, intervention program was well-designed, and the professionals who examined and guided all participants were very experienced. In addition, the one year duration of the study was long enough to allow meaningful conclusions to be drawn. Finally, the sample size was larger than in previous studies, with a wide age range of all participants, and included both children and adolescents. However, in our study we did not evaluate the nutritional value of the diet and the fatty acid intake in our participants; therefore, no correlations were made between nutritional intake and Irisin concentrations. 

We conclude that the decrease in BMI in children and adolescents with overweight or obesity following one year of implementation of a personalized, multi-disciplinary, personalized life-style intervention program of diet and exercise resulted in a significant reduction in Irisin concentrations, as well as an improvement in cardiometabolic risk factors. Further studies are required to determine the potential role of Irisin as a biomarker for monitoring the response to lifestyle interventions and for predicting the development of cardiometabolic risk factors.

## Figures and Tables

**Table 1:** Clinical characteristics of all subjects at baseline.

	Obese *N* = 211	Overweight *N* = 99	*p*-Value
Age (years)	10.7 (8.4–12.6)	10.3 (8.9–12.1)	0.934
Height (cm)	148.7 (135–159)	143.5 (137.2–157)	0.423
WC (cm)	86 (77–97.5)	77 (72–82)	**0.001**
HC (cm)	94 (83–104)	84 (77–91.5)	**0.001**
WHR	0.94 (0.89–0.99)	0.90 (0.85–0.97)	**0.014**
WHtR	0.59 (0.56–0.63)	0.53 (0.50–0.57)	**0.001**
SBP (mmHg)	112 (105–120)	110 (104–115)	**0.035**
DBP (mmHg)	65 (56–73)	60 (55–69)	**0.045**
Pubertal status			0.654
Prepubertal	102 (67.1)	50 (32.9)	
Pubertal	109 (69.0)	49 (31.0)	
Gender			**0.033**
Male	119 (73.5)	43 (26.4)	
Female	92 (62.2)	56 (37.8)	

Abbreviations: DBP, diastolic blood pressure; HC, hip circumference; SBP, systolic blood pressure; WC, waist circumference; WHR, waist-to-hip ratio; WHtR, waist-to-height ratio; continuous variables are presented as medians (interquartile range, IQR) and categorical as frequencies (percentages); *p* values were derived by comparisons between the three categories of BMI using *t*-test or Mann–Whitney; statistically significant associations are shown in bold.

**Table 2:** Clinical characteristics and biochemical, endocrinologic and body composition parameters in all subjects at initial and annual assessment.

	Initial Assessment *N* (%)	Annual Assessment *N* (%)	*p*-Value
Gender			
Male	162 (52.3)		
Female	148 (47.7)		
Pubertal status			
Prepubertal	152 (49.7)	97 (31.3)	
Pubertal	158 (50.3)	213 (68.7)	
BMI-percentile			**0.001**
Normal BMI		33 (10.6)	
Overweight	99 (31.9)	159 (51.3)	
Obesity	84 (27.1)	56 (18.1)	
>97th	127 (41.0)	61 (19.7)	
	**Mean (±SD)/** **Median (IQR)**	**Mean (±SD)/** **Median (IQR)**	
Age (years)	10.5 ± 2.9	11.6 ± 2.9	
Weight (kg)	59.8 ± 21.4	61.5 ± 20.9	
Height (cm)	146.4 ± 16.7	152.6 ± 16.1	
BMI (kg/m^2^)	25.9 (23.4–29.0)	24.6 (22.5–27.1)	**0.001**
WHtR	0.389 (0.318–0.457)	0.375 (0.322–0.448)	0.024 *
WC (cm)	82.0 (74.0–94.0)	82.0 (75.0–91.3)	0.897
HC (cm)	90.8 ± 15.1	92.6 ± 13.9	**0.002**
WHR	0.9 ± 0.1	0.9 ± 0.1	0.024 *
SBP (mmHg)	111.0 ± 12.4	112.5 ± 11.9	0.300
DBP (mmHg)	63.9 ± 11.2	67.1 ± 9.2	**0.006**
Glucose (mmol/L)	79.0 ± 8.7	80.6 ± 7.2	**0.006**
Insulin (mUI/mL)	14.1 (9.4–19.7)	13.2 (9.4–18.9)	0.435
HbA1C (%)	6.3 (5.1–5.4)	5.3 (5.1–5.4)	0.039 *
HOMA-IR	2.7 (1.8–3.9)	2.6 (1.8–3.9)	0.613
Urea (mmol/L)	29.3 ± 6.3	27.8 ± 6.1	**0.001**
Creatinine (μmol/L)	0.47 ± 0.1	0.53 ± 0.1	**0.001**
SGOT (IU/L)	23.0 (20.0–27.0)	21.0 (18.0–25.0)	**0.001**
SGPT (IU/L)	19.0 (15.0–24.0)	17.0 (14.0–21.0)	**0.001**
γ-GT (IU/L)	14.0 (11.0–18.0)	12.0 (10.0–16.0)	**0.001**
Cholesterol (nmol/L)	160.3 ± 28.3	151.2 ± 38.6	**0.001**
Triglycerides (mg/dL)	71.0 (53.0–105.0)	70.0 (50.0–97.0)	0.175
HDL (mmol/L)	49.7 ± 11.0	53.8 ± 13.5	**0.001**
LDL (mmol/L)	94.5 ± 22.7	87.1 ± 22.7	**0.001**
ApoA1 (g/L)	143.2 ± 22.1	142.3 ± 22.6	0.455
ApoΒ (g/L)	75.7 ± 18.6	71.1 ± 16.4	**0.001**
Lp(a) (g/L)	6.9 (2.5–14.1)	7.5 (2.9–15.0)	0.036 *
FT4 (pmol/L)	1.1 (1.0–1.2)	1.1 (1.0–1.2)	0.310
T3 (nmol/L)	147.0 (129.0–164.0)	140.0 (122.0–157.0)	**0.001**
TSH (mUI/L)	2.6 (2.0–3.6)	2.4 (1.9–3.3)	0.504
Anti-TG (IU/mL)	20.0 (20.0–20.0)	20.0 (20.0–20.0)	0.375
Anti-TPO (IU/mL)	10.0 (10.0–14.3)	10.0 (10.0–10.0)	0.507
IGF-I (μg/L)	264.0 (184.0–404.0)	367.0 (221.0–539.0)	**0.001**
IGFBP-3 (mg/L)	5.1 ± 1.1	5.4 ± 1.2	**0.001**
Total 25-OH-vitamin D (nmol/L)	22.2 ± 9.6	24.5 ± 9.6	**0.001**
ACTH (ng/L)	24.0 (16.5–35.1)	24.0 (16.6–32.6)	0.153
Cortisol (nmol/L)	14.1 (10.5–19.3)	12.7 (8.9–17.2)	**0.001**
SHBG (nmol/L)	40.2 (25.3–57.2)	40.9 (25.8–64.3)	0.173
hsCRP (mg/L)	0.36 ± 0.09	0.15 ± 0.01	0.040 *
Adiponectin (ng/mL)	16,408.5 (10,188.0–28,606.5)	20,585.5 (12,107.5–37,433.5)	**0.001**
Irisin (ng/mL)	391.4 (297.9–534.3)	212.6 (156.6–306.3)	**0.001**
FGF-21 (pg/mL)	23.9 (4.1–58.2)	27.7 (10.3–60.8)	0.087
FATP (%)	33.6 (30.9–38.1)	32.0 (28.8–35.7)	**0.001**
FATM (kg)	19.0 (14.3–24.8)	18.3 (14.4–23.6)	0.057
PMM (%)	36.6 ± 11.5	39.2 ± 11.7	**0.001**
BONEM (kg)	2.0 ± 0.6	2.1 ± 0.6	**0.001**
FFM (kg)	38.6 ± 12.0	41.3 ± 12.3	**0.001**

Abbreviations: ACTH, adrenocorticotropic hormone; Anti-TG, antibodies against thyroglobulin; Anti-TPO, thyroid peroxidase antibodies; ApoA1, apolipoprotein A1; ApoB, apolipoprotein B; BMI, body mass index; BONEM, bone mass; DBP, diastolic blood pressure; DHEA-s, dehydroepiandrosterone sulfate; T3, triiodothyronine; FT4, free thyroxine; FATM, fat mass; FATP, fat percentage; FFM, free-fat mass; FGF-21, Fibroblast growth factor 21; FSH, follicle stimulating hormone; γ-GT, serum gamma-glutamyltransferase; HbA1C, hemoglobin A1C; HbA1, hemoglobin A1; HC, hip circumference; HDL, high-density lipoprotein; HOMA-IR, homeostasis model assessment-insulin resistance; hsCRP, high sensitivity C-reactive protein; IGF1, insulin-like growth factor 1; IGF-BP3: IGF-binding protein 3 LDL, low-density lipoprotein; LH, luteinizing hormone; Lp(a), lipoprotein a; PMM, muscle mass percentage; PTH, parathormone; SGOT, serum glutamic oxaloacetic transaminase; SGPT: serum glutamic pyruvic transaminase; SBP, systolic blood pressure; SHBG, sex hormone-binding globulin; TAS, total oxidative status; TNF-α, tumor necrosis factor- alpha; TOS, total antioxidative status; TSH, thyroid stimulating hormone; WC, waist circumference; WHR, waist-to-hip ratio; WHtR, waist-to-height ratio; continuous variables are presented as means ±SD or with medians (interquartile range, IQR) and categorical ones as frequencies (percentages); *p* values were derived by paired samples *t*-test for normally distributed variables and Wilcoxon signed rank test for skewed variables; statistically significant associations are shown in bold; * not significant after Bonferroni adjustment for multiple testing.

**Table 3:** Correlation coefficients of Irisin and FGF-21 changes.

	Irisin Change	FGF-21 Change
BMI change	**−0.217 (0.001)**	0.077 (0.212)
BMI z-score change	**−0.201 (0.001)**	0.074 (0.228)
SBP change	−0.157 (0.131)	0.078 (0.477)
DBP change	−0.171 (0.100)	−0.072 (0.510)
WC change	**−0.423 (0.001)**	0.006 (0.944)
WHR change	**−0.234 (0.007)**	−0.119 (0.196)
CHOL change	−0.053 (0.382)	−0.061 (0.333)
HDL change	**0.232 (0.001)**	**−0.167 (0.007)**
TGL change	−0.039 (0.516)	0.056 (0.378)
Apo1 change	0.020 (0.743)	−0.039 (0.534)
ApoB change	−0.022 (0.716)	0.090 (0.152)
Lp(a) change	0.005 (0.936)	−0.088 (0.162)
HOMA-IR change	−0.111 (0.055)	−0.048 (0.452)
FATP change	−0.061 (0.369)	−0.062 (0.385)
PMM change	−0.069 (0.308)	**0.207 (0.003)**
BONEM change	−0.029 (0.673)	**0.209 (0.003)**
VitD change	0.013 (0.828)	−0.042 (0.504)
PTH change	**−0.151 (0.012)**	0.035 (0.575)
Leptin change	0.021 (0.727)	−0.039 (0.528)
Adiponectin change	**−0.161 (0.007)**	0.043 (0.488)
TMI change	**−0.192 (0.001)**	0.050 (0.420)

Abbreviations: Apo1, apolipoprotein 1; ApoB, apolipoprotein B; BMI, body mass index; BONEM, bone mass; CHOL, cholesterol; DBP, diastolic blood pressure; FATP, fat percentage; FGF-21, fibroblast growth factor 21; HDL, high-density lipoprotein; HOMA-IR, homeostatic model assessment-insulin resistance; Lp(a), lipoprotein-a; PTH, parathormone; PMM, muscle mass percentage; SBP, systolic blood pressure; TGL, triglycerides; TMI, tri-ponderal mass index; VitD, total 25-OH Vitamin D; WC, waist circumference; WHR, waist-to-hip ratio; variables are presented as Pearson’s *r* and spearman’s rho correlation coefficients (*p* value); statistically significant associations are shown in bold.

**Table 4:** Stratified correlations of Irisin and FGF-21 changes and BMI changes according to gender, pubertal status, and BMI percentiles.

	Irisin Change	FGF-21 Change
Male		
BMI change	**−0.302 (0.001)**	0.109 (0.205)
BMI z-score change	**−0.277 (0.001)**	0.097 (0.258)
Female		
BMI change	**−0.302 (0.001)**	0.109 (0.205)
BMI z-score change	**−0.277 (0.001)**	0.097 (0.258)
Prepubertal subjects (baseline)		
BMI change	−0.139 (0.100)	0.084 (0.339)
BMI z-score change	−0.102 (0.229)	0.050 (0.569)
Pubertal subjects (baseline)		
BMI change	**−0.265 (0.001)**	0.068 (0.446)
BMI z-score change	**−0.264 (0.001)**	0.085 (0.337)
Same pubertal stage		
BMI change	**−0.275 (0.001)**	0.122 (0.095)
BMI z-score change	**−0.231 (0.001)**	0.133 (0.067)
Enter puberty		
BMI change	−0.172 (0.294)	−0.060 (0.720)
BMI z-score change	−0.182 (0.266)	0.023 (0.893)
Overweight (baseline)		
BMI change	−0.192 (0.069)	0.042 (0.703)
BMI z-score change	−0.262 (0.012)	0.005 (0.961)
Obese (baseline)		
BMI change	**−0.407 (0.001)**	0.105 (0.368)
BMI z-score change	**−0.388 (0.001)**	0.061 (0.602)
>97th percentile (baseline)		
BMI change	−0.140 (0.130)	0.027 (0.786)
BMI z-score change	−0.068 (0.465)	0.053 (0.592)

Abbreviations: BMI, body mass index; FGF-21, fibroblast growth factor 2; variables are presented as Pearson’s r and spearman’s rho correlation coefficients (*p* value); statistically significant associations are shown in bold.

**Table 5:** β regression coefficients for the Irisin change.

	Irisin Change	
	**β (95% CI)**	***p*** **-Value**
BMI Z-score change	92.78 (−100.05 to 285.61)	0.342
WHR change	−465.55 (−918.40 to 12.70)	**0.044**
HDL change	1.66 (−3.05 to 6.38)	0.486
PTH change	−2.28 (−6.18 to 1.61)	0.248
Adiponectin change	−0.002 (−0.004 to 0.001)	0.199
TMI change	−39.26 (−132.23 to 53.72)	0.405
Age	−3.16 (−27.81 to 21.48)	0.800
Gender (girls vs. boys)	−29.84 (−145.76 to 86.08)	0.611
Pubertal Stage (Pubertal vs. pre-pubertal	17.28 (−114.34 to 148.89)	0.795

Abbreviations: BMI Z-score, Body mass index Z-score; HDL, high-density lipoprotein; PTH, parathormone; TMI, tri-ponderal mass index; WHR, waist-to-hip ratio; variables are presented as β-values, 95% confidence intervals (CI); statistically significant associations are shown in bold.

## Supplementary Materials

The following are available online at https://www.mdpi.com/article/10.3390/nu13041274/s1, Table S1: Correlation coefficients of variables at baseline and at 12 months.

## References

[1] NCD Risk Factor Collaboration (2017). Worldwide trends in body-mass index, underweight, overweight, and obesity from 1975 to 2016: A pooled analysis of 2416 population-based measurement studies in 128·9 million children, adolescents, and adults. Lancet.

[2] World Health Organization(WHO) (2017). Commission on Ending Childhood Obesity. https://www.who.int/end-childhood-obesity/en/.

[3] World Health Organization(WHO) (2018). Fact Sheets. Obesity and Overweigh. https://www.who.int/news-room/fact-sheets/detail/obesity-and-overweight.

[4] Dobbs R., Sawers C., Thompson F., Manyika J., Woetzel J., Child P., McKenna S., Spatharou A. (2014). Overcoming Obesity: An Initial Economic Analysis. https://www.mckinsey.com/~/media/mckinsey/business%20functions/economic%20studies%20temp/our%20insights/how%20the%20world%20could%20better%20fight%20obesity/mgi_overcoming_obesity_full_report.ashx.

[5] Spinelli A., Buoncristiano M., Kovacs V.A., Yngve A., Spiroski I., Obreja G., Starc G., Pérez N., Rito A.I., Kunešová M. (2019). Prevalence of Severe Obesity among Primary School Children in 21 European Countries. Obesity.

[6] Saponaro C., Gaggini M., Carli F., Gastaldelli A. (2015). The subtle balance between lipolysis and lipogenesis: A critical point in metabolic homeostasis. Nutrients.

[7] Fasshauer M., Blu M. (2015). Adipokines in health and disease. Trends Pharmacol. Sci..

[8] Boström P., Wu J., Jedrychowski M.P., Korde A., Ye L., Lo J.C., Rasbach K., Boström E.A., Choi J.H., Long J.Z. (2012). A PGC1a dependent myokine that derives browning of white fat and thermogenesis. Nature.

[9] Roca-rivada A., Castelao C., Senin L., Landrove M., Baltar J., Crujeiras B., Seoane L., Casanueva F., Pardo M. (2013). FNDC5/Irisin Is Not Only a Myokine but Also an Adipokine. PLoS ONE.

[10] Perakakis N., Triantafyllou G.A., Fernández-Real J.M., Huh J.Y., Park K.H., Seufert J., Mantzoros C.S. (2017). Physiology and role of irisin in glucose homeostasis. Nat. Rev. Endocrinol..

[11] Binay Ç., Paketçi C., Güzel S., Samancı N. (2017). Serum Irisin and Oxytocin Levels as Predictors of Metabolic Parameters in Obese Children. J. Clin. Res. Pediatr. Endocrinol..

[12] Catli G., Kume T., Tuhan H.U., Anik A., Calan O.G., Bober E., Abaci A. (2016). Relation of Serum Irisin Level with Metabolic and Antropometric Parameters in Obese Children. J. Diabetes Complicat..

[13] Sesti G., Andreozzi F., Fiorentino T.V., Mannino G.C., Sciacqua A., Marini M.A., Perticone F. (2014). High circulating irisin levels are associated with insulin resistance and vascular atherosclerosis in a cohort of nondiabetic adult subjects. Acta Diabetol..

[14] Seppä S., Tenhola S., Voutilainen R. (2019). Fibroblast Growth Factor 21, Adiponectin, and Irisin as Markers of Unfavorable Metabolic Features in 12-Year-Old Children. J. Endocr. Soc..

[15] Park K.H., Zaichenko L., Brinkoetter M., Thakkar B., Sahin-efe A., Joung K.E., Tsoukas M., Geladari E.V., Huh J.Y., Dincer F. (2013). Circulating Irisin in Relation to Insulin Resistance and the Metabolic Syndrome. J. Clin. Endocrin. Metab..

[16] Elizondo-montemayor L., Silva-platas C., Torres-quintanilla A., Rodríguez-lópez C., Ruiz-esparza G.U., Reyes-mendoza E., Garcia-rivas G. (2017). Association of Irisin Plasma Levels with Anthropometric Parameters in Children with Underweight, Normal Weight, Overweight, and Obesity. Biomed. Res. Int..

[17] Dozio E., Vianello E., Sitzia C., Ambrogi F., Benedini S., Gorini S., Rampoldi B., Rigolini R., Tacchini L., Marco M. (2020). Circulating Irisin and esRAGE as Early Biomarkers of Decline of Metabolic Health. J. Clin. Med..

[18] Nishimura T., Nakatake Y., Konishi M., Itoh N. (2000). Identification of a novel FGF, FGF-21, preferentially expressed in the liver. Biochim. Biophys. Acta Gene Struct. Expr..

[19] Inagaki T. (2015). Research Perspectives on the Regulation and Physiological Functions of FGF21 and its Association with NAFLD. Front. Endocrinol..

[20] Ding X., Boney-montoya J., Owen B.M., Bookout A.L., Coate C., Mangelsdorf D.J., Kliewer S.A. (2013). βKlotho Is Required for Fibroblast Growth Factor 21 Effects On Growth and Metabolism. Cell Metab..

[21] Smith R., Duguay A., Bakker A., Li P., Weiszmann J., Thomas M.R., Alba B.M., Wu X., Gupte J., Yang L. (2013). FGF21 Can Be Mimicked In Vitro and In Vivo by a Novel Anti-FGFR1c/β-Klotho Bispecific Protein. PLoS ONE.

[22] Markan K.R., Naber M.C., Ameka M.K., Anderegg M.D., Mangelsdorf D.J., Kliewer S.A., Mohammadi M., Potthoff M.J. (2014). Circulating FGF21 is liver derived and enhances glucose uptake during refeeding and overfeeding. Diabetes.

[23] Fisher M., Kleiner S., Douris N., Fox E.C., Mepani R.J., Verdeguer F., Wu J., Kharitonenkov A., Flier J.S., Maratos-flier E. (2012). FGF21 regulates PGC-1 a and browning of white adipose tissues in adaptive thermogenesis. Genes Dev..

[24] Lin Z., Gong Q., Wu C., Yu J., Lu T., Pan X., Lin S., Li X. (2012). Dynamic Change of Serum FGF21 Levels in Response to Glucose Challenge in Human. J. Clin. Endocrinol. Metab..

[25] Li X. (2019). The FGF metabolic axis. Front. Med..

[26] Hanssen M.J.W., Broeders E., Samms R.J., Vosselman M.J., van der Lans A.A.J.J., Cheng C.C., Adams A.C., van Marken Lichtenbelt W.D., Schrauwen P. (2015). Serum FGF21 levels are associated with brown adipose tissue activity in humans. Sci. Rep..

[27] Ibarra-Reynoso L.D.R., Pisarchyk L., Pérez-Luque E.L., Garay-Sevilla M.E., Malacara J.M. (2015). Dietary restriction in obese children and its relation with eating behavior, fibroblast growth factor 21 and leptin: A prospective clinical intervention study. Nutr. Metab..

[28] Reinehr T., Karges B., Meissner T., Wiegand S., Fritsch M., Holl R.W., Woelfle J. (2015). Fibroblast growth factor 21 and fetuin-A in obese adolescents with and without type 2 diabetes. J. Clin. Endocrinol. Metab..

[29] Wang Q., Yuan J., Yu Z., Lin L., Jiang Y., Cao Z., Zhuang P., Whalen M.J., Song B., Wang X.J. (2017). FGF21 Attenuates High-Fat Diet-Induced Cognitive Impairment via Metabolic Regulation and Anti-inflammation of Obese Mice. Mol. Neurobiol..

[30] Gaich G., Chien J.Y., Fu H., Glass L.C., Deeg M.A., Holland W.L., Kharitonenkov A., Bumol T., Schilske H.K., Moller D.E. (2013). The effects of LY2405319, an FGF21 Analog, in obese human subjects with type 2 diabetes. Cell Metab..

[31] Baek J., Nam H., Rhie Y., Lee K., Lee K. (2017). Serum FGF21 Levels in Obese Korean Children and Adolescents. J. Obes. Metab. Syndr..

[32] Itoh N. (2014). FGF21 as a hepatokine, adipokine, and myokine in metabolism and diseases. Front. Endocrinol..

[33] Tanajak P., Pongkan W., Chattipakorn S.C., Chattipakorn N. (2018). Increased plasma FGF21 level as an early biomarker for insulin resistance and metabolic disturbance in obese insulin-resistant rats. Diabetes Vasc. Dis. Res..

[34] Reinehr T., Woelfle J., Wunsch R., Roth C.L. (2012). Fibroblast Growth Factor 21 (FGF-21) and Its Relation to Obesity, Metabolic Syndrome, and Nonalcoholic Fatty Liver in Children: A Longitudinal Analysis. J. Clin. Endocrinol. Metab..

[35] Löffler D., Müller U., Scheuermann K., Friebe D., Gesing J., Bielitz J., Erbs S., Landgraf K., Wagner I.V., Kiess W. (2015). Serum irisin levels are regulated by acute strenuous exercise. J. Clin. Endocrinol. Metab..

[36] Viitasalo A., Ågren J.T., Venäläinen J., Pihlajamäki J., Jääskeläinen A., Korkmaz A., Atalay M., Lakka T.A. (2015). Association of plasma fatty acid composition with plasma irisin levels in normal weight and overweight/obese children. Pediatr. Obes..

[37] Reinehr T., Elfers C., Lass N., Roth C.L. (2015). Irisin and its relation to insulin resistance and puberty in obese children: A longitudinal analysis. J. Clin. Endocrinol. Metab..

[38] Palacios-Gonzalez B., Vadillo-Ortega F., Polo-Oteyza E., Sanchez T., Ancira-Moreno M., Romero-Hidalgo S., Meraz N., Antuna-Puente B. (2015). Irisin Levels Before and After Physical Activity Among School-Age Children with Different BMI: A Direct Relation with Leptin. Obesity.

[39] Bluher S., Panagiotou G., Petroff D., Markert J., Wagner A., Klemm T., Filippaios A., Keller A., Mantzoros C.S. (2014). Effects of a 1-Year Exercise and Lifestyle Intervention on Irisin, Adipokines, and Inflammatory Markers in Obese Children. Obesity.

[40] Cole T.J., Lobstein T. (2012). Extended international (IOTF) body mass index cut-offs for thinness, overweight and obesity. Pediatr. Obes..

[41] Güngör N.K. (2014). Overweight and obesity in children and adolescents. JCRPE J. Clin. Res. Pediatr. Endocrinol..

[42] Kaur Y., de Souza R.J., Gibson W.T., Meyre D. (2017). A systematic review of genetic syndromes with obesity. Obes. Rev..

[43] World Health Organization(WHO) (2008). Waist Circumference and Waist–Hip Ratio: Report of a WHO Expert Consultation. https://apps.who.int/iris/handle/10665/44583.

[44] Genitsaridi S.M., Giannios C., Karampatsou S., Papageorgiou I., Papadopoulos G., Farakla I., Koui E., Georgiou A., Romas S., Terzioglou E. (2020). A comprehensive multidisciplinary management plan is effective in reducing the prevalence of overweight and obesity in childhood and adolescence. Horm. Res. Paediatr..

[45] Birch L., Perry R., Hunt L.P., Matson R., Chong A., Beynon R., Shield J.P.H. (2019). What change in body mass index is associated with improvement in percentage body fat in childhood obesity?. A Meta Regres. BMJ Open.

[46] Conway L.J., Ingwersen A.M. (2004). Accuracy of Dietary Recall Using the USDA Five-Step Multiple-Pass Method in Men: An Observational Validation Study. J. Am. Diet. Assoc..

[47] USDA and CNPP (2011). MyPlate Background. https://www.myplate.gov.

[48] Nigro E., Scudiero O., Ludovica M., Polito R., Schettino P., Grandone A., Perrone L., Miraglia E., Giudice D., Daniele A. (2017). Cytokine Adiponectin profile and Irisin expression in Italian obese children: Association with insulin-resistance. Cytokine.

[49] Hedblad B., Calling S., Berglund G., Janzon L. (2006). Sex differences in the relationships between BMI, WHR and incidence of cardiovascular disease: A population-based cohort study. Int. J. Obes..

[50] Rönnecke E., Vogel M., Bussler S., Grafe N., Jurkutat A., Schlingmann M., Koerner A., Kiess W. (2019). Age- and Sex-Related Percentiles of Skinfold Thickness, Waist and Hip Circumference, Waist-to-Hip Ratio and Waist-to-Height Ratio: Results from a Population-Based Pediatric Cohort in Germany (LIFE Child ). Obes. Facts.

[51] Singhal V., Lawson E.A., Ackerman K.E., Fazeli P.K., Clarke H., Lee H., Eddy K., Marengi D.A., Derrico N.P., Bouxsein M.L. (2014). Irisin Levels Are Lower in Young Amenorrheic Athletes Compared with Eumenorrheic Athletes and Non-Athletes and Are Associated with Bone Density and Strength Estimates. PLoS ONE.

[52] Pardo M., Crujeiras A.B., Amil M., Aguera Z., Jiménez-murcia S., Baños R., Botella C., De Torre R., Estivill X., Fagundo A.B. (2014). Association of Irisin with Fat Mass, Resting Energy Expenditure, and Daily Activity in Conditions of Extreme Body Mass Index. Int. J. Endocrinol..

[53] De Meneck F., De Souza L.V., Oliveira V., Franco C. (2018). High Irisin Levels in Overweight/Obese Children and Its Positive Correlation with Metabolic Profile, Blood Pressure and Endothelial Progenitor Cells. Nutr. Metab. Cardiovasc. Dis..

[54] Hu W., He J., Fu W., Wang C., Yue H., Gu J., Zhang H., Zhang Z. (2018). Fibroblast Growth Factor 21 is Associated with Bone Mineral Density, but not with Bone Turnover Markers and Fractures in Chinese Postmenopausal Women. J. Clin. Densitom..

[55] Hanks L.J., Gutiérrez O.M., Bamman M.M., Ashraf A., Mccormick K.L., Casazza K. (2015). Circulating levels of fibroblast growth factor-21 increase with age independently of body composition indices among healthy individuals. J. Clin. Transl. Endocrinol..

[56] Ruiz-padilla A.J., Morales-hernandez G., Ruiz-noa Y., Josabad A., Lazo-de-la-vega-monroy M.L., Preciado-puga M.C., Rangel-salazar R., Ibarra-reynoso L.R. (2019). Association of the 3′ UTR polymorphism (rs11665896) in the FGF21 gene with metabolic status and nutrient intake in children with obesity. J. Pediatr. Endocrinol. Metab..

[57] Lips M.A., Groot G.H., De Berends F.J., Wiezer R., Wagensveld B.A., Van Swank J., Luijten A., Van Dijk K.W., Pijl H., Jansen P.L.M. (2014). Calorie restriction and Roux-en-Y gastric bypass have opposing effects on circulating FGF21 in morbidly obese subjects. Clin. Endocrinol..

[58] Mashili F.L., Ramaiya K., Lutale J., Njelekela M., Francis F., Zierath J., Krook A. (2018). Adiposity Is a Key Correlate of Circulating Fibroblast Growth Factor-21 Levels in African Males with or without Type 2 Diabetes Mellitus. J. Obes..

