# Supplementary material for: The Effect of a Life-Style Intervention Program of Diet and Exercise on Irisin and FGF-21 Concentrations in Children and Adolescents with Overweight and Obesity
Source: Nutrients. 2021 Apr 13;13(4):1274. doi: 10.3390/nu13041274 (PMC8070027; doi:10.3390/nu13041274)
Supplement: Supplementary file 1 [file nutrients-13-01274-s001.pdf]

nutrients

MDPI

Supplemental Table 1: Correlation coefficients of variables at baseline and at 12 months

	Initial assessment		Annual assessment	
	**Irisin**	**FGF-21**	**Irisin**	**FGF-21**
**BMI (kg/m²)**	0.028 (0.628)	**0.235 (0.001)**	-0.034 (0.553)	**0.202 (0.001)**
**BMI z-score**	0.025 (0.671)	0.097 (0.093)	-0.062 (0.283)	**0.133 (0.027)**
**SBP (mmHg)**	-0.064 (0.359)	0.134 (0.056)	-0.027 (0.747)	**0.210 (0.016)**
**DBP (mmHg)**	-0.097 (0.168)	0.074 (0.296)	-0.072 (0.391)	0.021 (0.808)
**WC (cm)**	-0.077 (0.218)	**0.272 (0.001)**	-0.113 (0.170)	-0.018 (0.838)
**WHR**	0.011 (0.856)	0.095 (0.129)	0.017 (0.777)	**-0.127 (0.037)**
**Cholesterol (mmol/L)**	0.031 (0.595)	-0.107 (0.068)	-0.059 (0.315)	-0.061 (0.315)
**HDL (mmol/L)**	0.012 (0.838)	**-0.182 (0.002)**	0.033 (0.577)	0.070 (0.252)
**TGL (mg/dL)**	0.024 (0.684)	**0.243 (0.001)**	-0.046 (0.434)	**0.121 (0.047)**
**ApoA1 (g/dL)**	0.012 (0.843)	-0.076 (0.192)	-0.089 (0.126)	**0.148 (0.015)**
**ApoB (g/dL)**	-0.004 (0.940)	-0.010 (0.860)	**-0.149 (0.009)**	0.069 (0.252)
**Lp(a) (g/dL)**	-0.098 (0.091)	0.012 (0.841)	-0.007 (0.910)	**0.293 (0.001)**
**HOMA-IR**	0.017 (0.765)	**0.221 (0.001)**	0.033 (0.574)	**0.227 (0.001)**
**FATP (%)**	-0.003 (0.969)	**0.134 (0.043)**	0.064 (0.274)	-0.094 (0.126)
**PMM (kg)**	0.054 (0.416)	**0.206 (0.002)**	-0.037 (0.526)	**0.183 (0.003)**
**BONEM (kg)**	0.064 (0.335)	**0.197 (0.003)**	0.038 (0.506)	**0.175 (0.004)**
**VitD (nmol/L)**	0.111 (0.055)	**-0.065 (0.263)**	-0.034 (0.553)	**0.202 (0.001)**
**PTH (ng/L)**	**-0.175 (0.003)**	**0.171 (0.003)**	-0.062 (0.283)	**0.133 (0.027)**
**Leptin (pg/mL)**	0.087 (0.132)	0.106 (0.068)	-0.027 (0.747)	**0.210 (0.016)**
**Adiponectin (ng/mL)**	0.002 (0.973)	-0.049 (0.398)	-0.072 (0.391)	0.021 (0.808)
**TMI (kg/m³)**	0.032 (0.585)	0.101 (0.081)	-0.113 (0.170)	-0.018 (0.838)

Abbreviations: Apo1, apolipoprotein 1; ApoB, apolipoprotein B; BMI, body mass index; BONEM, bone mass; DBP, diastolic blood pressure; FATP, fat percentage; FGF-21, fibroblast growth factor 21; HDL, high-density lipoprotein; HOMA-IR:, homeostatic model assessment-Insulin Resistance; Lp(a), lipoprotein-a; PMM, muscle mass percentage; SBP, systolic blood pressure; TG, triglycerides; TMI, tri-ponderal mass index; VitD, total 25-OH vitamin D; WC, waist circumference; WHR, waist-to-hip ratio; variables are presented as Pearson’s r and spearman's rho correlation coefficients (p value); statistically significant associations are shown in bold.
